# A systematic review on the effectiveness of implementation strategies to postpone elective caesarean sections to ≥ 39 + (0–6) weeks of gestation

**DOI:** 10.1186/s13643-021-01718-1

**Published:** 2021-06-14

**Authors:** Barbara Prediger, Anahieta Heu-Parvaresch, Stephanie Polus, Stefanie Bühn, Edmund A. M. Neugebauer, Pieper Dawid

**Affiliations:** 1 Institute for Research in Operative Medicine, Witten/Herdecke University, Ostmerheimer Str. 200, Haus 38, 51109 Cologne, Germany; 2 Institute for Medical Sociology, Health Services Research, and Rehabilitation Science, The University Hospital of Cologne (AöR), Cologne, Germany; 3 Institute for Medical Information Processing, Biometry and Epidemiology, LMU Munich, Munich, Germany; 4 Pettenkofer School of Public Health, LMU Munich, Munich, Germany; 5 Faculty of Health, Brandenburg Medical School — Theodor Fontane, Campus Neuruppin, Fehrbelliner Str.38, 16816 Neuruppin, Germany

**Keywords:** Elective caesarean section, Term birth, Gestational age, Implementation strategies, Guidelines into practice

## Abstract

**Background:**

Caesarean sections often have no urgent indication and are electively planned. Research showed that elective caesarean section should not be performed until 39 + (0–6) weeks of gestation to ensure best neonatal and maternal health if there are no contraindications. This was recommended by various guidelines published in the last two decades. With this systematic review, we are looking for implementation strategies trying to implement these recommendations to reduce elective caesarean section before 39 + (0–6) weeks of gestation.

**Methods:**

We performed a systematic literature search in MEDLINE, EMBASE, CENTRAL, and CINAHL on 3rd of March 2021. We included studies that assessed implementation strategies aiming to postpone elective caesarean section to ≥ 39 + (0–6) weeks of gestation. There were no restrictions regarding the type of implementation strategy or reasons for elective caesarean section. Our primary outcome was the rate of elective caesarean sections before 39 + (0–6) weeks of gestation. We used the ROBINS-I Tool for the assessment of risk of bias. We did a narrative analysis of the results.

**Results:**

We included 10 studies, of which were 2 interrupted time series and 8 before-after studies, covering 205,954 elective caesarean births. All studies included various types of implementation strategies. All implementation strategies showed success in decreasing the rate of elective caesarean sections performed < 39 + (0–6) weeks of gestation. Risk difference differed from − 7 (95% CI − 8; − 7) to − 45 (95% CI − 51; − 31). Three studies reported the rate of neonatal intensive care unit admission and showed little reduction.

**Conclusion:**

This systematic review shows that all presented implementation strategies to reduce elective caesarean section before 39 + (0–6) weeks of gestation are effective. Reduction rates differ widely and it remains unclear which strategy is most successful. Strategies used locally in one hospital seem a little more effective. Included studies are either before-after studies (8) or interrupted time series (2) and the overall quality of the evidence is rather low. However, most of the studies identified specific barriers in the implementation process. For planning an implementation strategy to reduce elective caesarean section before 39 + (0–6) weeks of gestation, it is necessary to consider specific barriers and facilitators and take all obstetric personal into account.

**Systematic review registration:**

PROSPERO CRD42017078231

**Supplementary Information:**

The online version contains supplementary material available at 10.1186/s13643-021-01718-1.

## Background

The rates of caesarean section (CS) in high-income countries is currently about 30% of all births [1]. The World Health Organization (WHO) states that there is no medical reason for a higher rate of CSs than 10–15%, though [2, 3]. One of the most common reasons for performing an elective CS is a previous CS [4]. Even though vaginal birth after CS (VBAC) is recommended for the majority of women, studies showed that in the UK only 50% and in the USA only 10 % of women undergo VBAC [5, 6]. Reasons for retentions from VBAC are that in following pregnancies, especially in the late term (≥ 39 + (0–6) weeks of gestation (WG)), risks of scar rupture in women with a scarred uterus increase or lead to emergency CS [7]. Studies on emergency repeated CS assume severe bleeding needing transfusion and even higher mortality, leading to the assumption that planning CS at early term (37 + 0 to 38 + 6 WG) is safer [8, 9]. On the other hand, early-term elective CS increases the risk of respiratory diseases in neonates and admission to the neonatal intensive care unit (NICU) [10].

In the last two decades, numerous guidelines and recommendations on CS in general and on timing of elective CS in specific have been published, while the National Institute for Health and Care Excellence (NICE) was the first in publishing their first edition of the guideline “Caesarean Section” in 2004 [11]. NICE “Birth after previous caesarean birth” by the Royal College for Obstetricians and Gynecologists (RCOG), “Timing of elective Caesarean Section at term” by the Royal Australian and New Zealand College of Obstetricians and Gynaecologists (RANZCOG) and “Die Sectio Caesarea” by the German Society of Gynecologists and Obstetricians (DGGG) examined if early-term CS increases respiratory morbidity of the neonate. All recommend performing uncomplicated elective CS not before the 39 + (0–6) WG [12–14]. In their committee opinions, 764 and 765 the American College of Obstetricians and Gynecologists (ACOG) recommends not performing any indicated deliveries (both induction of labour and CS) before the 39 + (0–6) WG in uncomplicated pregnancies [15, 16]. A recent systematic review and meta-analysis of 30 studies assessing the timing of elective CS has shown that risks for the mother and the neonate are lowest in the 39 + (0–6) WG. Risks for neonates are decreasing from 37 + (0–6) WG onwards and there seems to be no increase in risks for mothers until the 39 + 0–6 WG [17]. This shows that the recommendations given before still last. Nevertheless, it is not fully integrated in clinical practice yet.

However, the main issue is the successful integration of a guideline into practice [18, 19]. Research says that generally ineffective strategies to change physician practice are written information and continuous medical education [20, 21]. Effective strategies to change physician practice are academic detailing and multifaceted intervention (e.g., educational material combined with audit and feedback) though [22, 23]. Audit and feedback alone, as well as local opinion leaders and continuous quality improvement strategies, have mixed effects [24–27]. Additionally, the success of implementation of guidelines depends on the clinical setting. Each medical specialty has its own organizational and cultural characteristics. It is necessary to identify barriers and facilitators to improve effectiveness of guideline implementation [28].

### Objectives

We performed a systematic review of the literature to evaluate the effect of implementation strategies to shift elective CS to ≥ 39 + 0–6 WG.

## Methods

### Protocol and registration

We registered our review at PROSPERO (CRD42020166569) and followed the Preferred Reporting Items for Systematic Reviews and Meta-Analyses (PRISMA) while writing that systematic review [29].

### Eligibility criteria

We included studies assessing any implementation strategy aiming to shift elective CS at term (≥ 37 + 0 WG) from early term (37 + 0–38 + 6 WG) to late term (≥ 39 + (0–6) WG), regardless if it was first CS, repeated CS, singleton, or multiple pregnancies. Implementation strategies could be guidelines, education, rules, laws, policies, quality improvement, or any other intervention promoting the delay of elective CS. The intervention could be an international, national, regional, or just hospital-based strategy. We did not restrict the intervention to any duration or time of implementation. Interventions could be directed to any health care professionals but also to the pregnant women. Moreover, studies assessing the influence of the publication of a guideline in general were included. Comparators were no intervention or other types of implementation strategies. As randomized trials are rarely available to evaluate effects of health systems implementation strategies, according to the Cochrane Effective Practice and Organisation of Care, we considered a broader range of study designs [30]. We included (quasi-) randomized trials, non-randomized controlled trials, cohort studies, (controlled) before-after studies, and interrupted time series studies with or without control group. We did not make any restrictions regarding the language and publication date.

### Outcomes

The primary outcome was elective CS rate performed early term (before the 39 + (0–6) WG). We also assessed the rate of admissions to the NICU. All outcomes were collected as absolute numbers.

### Information sources

We searched MEDLINE, EMBASE, CENTRAL, and CINAHL on 3rd of March 2021. We did not restrict the search to any language or publication date. To identify grey literature, we searched Google Scholar additionally on 16 of March 2021. We also contacted authors of guidelines to identify studies we did not found by our systematic search.

We also checked the references of included studies, guidelines, and systematic reviews and if necessary contacted authors for additional data.

### Search strategy

The search strategy was developed using MeSH terms and text words by a librarian applying the PRESS checklist [31]. The search strategies are available in appendix A.

### Study selection

Records identified through the searches were added to an Endnote X9 database and duplicates were removed. Two reviewers assessed the relevance of the identified titles and abstracts independently. The same 2 reviewers assessed the studies, which were included for full text review again independently. We discussed differences until a consensus was found or a third reviewer was included.

### Data collection

Data was collected in an a priori-piloted extraction table by one reviewer, and the other reviewer monitored all entries for completeness and accuracy. We extracted data directly in an excel sheet.

### Data items

We extracted following study characteristics: Author, publication year, region, setting, data base, study design, recruitment period, inclusion and exclusion criteria of the patients, intervention characteristics, and outcomes. We oriented ourselves by the TIDieR checklist to set up a framework of reporting the interventions [32].

### Risk of bias assessment

For RCTs, we would have used the Cochrane Risk of Bias Tool [33]. For cohort studies, (controlled) before-after studies, and interrupted time series with or without control group, we used the ROBINS-I tool [34]. Two reviewers independently assessed risk of bias. We discussed differences until we found a consensus.

### Data synthesis

Due to multiple intervention types and very heterogenic study characteristics, we could not synthesize data in any meta-analysis. We condensed the results in a structured narrative analysis by using the Synthesis Without Meta-Analysis guidance (SWiM) [35]. We reported effects of single studies and the range of results and vote counted for effective/no difference/harm. We checked for similarities and differences in the description of the intervention and defined “categories” of implementation strategies for better comparability and interpretation of findings. By “category”, we mean the type of intervention (e.g., policy or guideline) and the level (e.g., regional or local hospital setting). Additionally, we categorized strategies into written information, continuous medical education, audit and feedback, local opinion leaders, (continuous) quality improvement strategies, academic detailing, or multifaceted intervention if possible [36]. We reported risk differences and odds ratios, both unadjusted or adjusted, if available. If not reported, we calculated the risk difference associated with implementation of the intervention as percent and the 95% confidence interval, if possible. If more than one time point was reported, we extracted the outcomes with the longest follow-up time. We also displayed the results graphically showing the studies on a timeline while considering the date of guideline publication.

### Risk of bias across studies

#### Publication bias

We could not create a funnel plot to inspect asymmetry of the results visually as we did not include enough studies.

#### Selective reporting within studies

If available, study protocols were checked and compared with reporting in studies. We contacted the authors of the studies to detect protocols if not stated otherwise. We also checked studies for their sources of funding.

### Additional analyses

We planned to perform subgroup analysis for the same intervention category (e.g., local hospital policy), but we did not perform any additional analyses as data was not sufficient to do so.

## Results

### Study selection

**Fig. 1:**
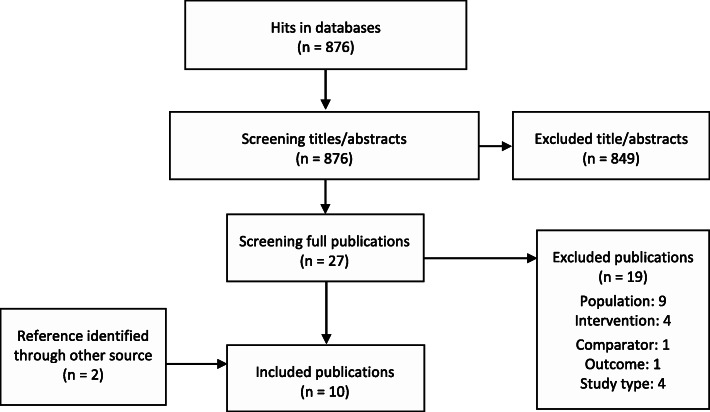
Study selection flow diagram

We identified 876 hits in the databases after duplicate removal (Fig. 1). We screened 27 publications in full text of which we included 8 in the review. We identified 2 additional publications by screening the reference lists of a systematic review. The references from the guidelines, the search in Google Scholar, and asking clinical experts (authors of the guidelines stated above and authors of the included studies) about studies, we have not identified resulted in no additional inclusions. The included and excluded (with reason) studies are presented in appendix B.

### Study characteristics

Of the included studies, 8 studies were before-after studies [37–44] and 2 studies were interrupted time series [45, 46]. We identified 2 studies conducted in Canada [38, 46], 3 in the USA [37, 43, 44], 2 in the UK [40, 45], 2 in Australia [39, 41], and one in the Netherlands [42]. The study from Snowden et al. resulted in another publication by Muoto et al. and is an additional analysis [37, 47]. We used the first publication by Snowden et al. and added outcome data from the subsequent publication. All studies included women with elective CS which was safe for mother and neonate to be postponed from 37 + 0–38 + 6 WG to ≥ 39 + (0–6) WG. The included studies covered 205,954 elective CS. Reporting of eligibility criteria for inclusion in the individual studies differed a lot. All studies stated that postponing of elective CS from 37 + 0–38+6 WG to ≥ 39 + (0–6) WG needed to be safe for mother and neonate. However, the description of safety varied, e.g., Tanger et al. excluded women with a medical history or pregnancy-related complications (e.g., preeclampsia, maternal infection, (suspicion of) foetal distress, severe birth defects of the foetus, maternal gestational diabetes, or diabetes mellitus) while Nicoll et al. only claimed to include all deliveries with elective CS at term and delaying delivery would be without any risk to the mother or foetus [40, 42]. Dunn et al. only included women with elective repeat CS [38]. Inclusion criteria are described in detail in Table 1.

**Table 1:** Inclusion/exclusion criteria and patient characteristics

Study	Inclusion/Exclusion criteria	Patient characteristics ***Control/Intervention:***
Allen 2020	Inclusion criteria: All births ≥ 37 WG. Exclusion criteria: Paid by other than Medicaid or private insurance.	NR
Dunn 2013	Inclusion criteria: All ERCS ≥ 37 WG in “Low-risk women”. Exclusion criteria: Women, who were in labour, had a medical history or pregnancy-related complications.	ERCS n, 459/497
Gurol-Urganci 2014	Inclusion criteria: All ECS ≥ 37 WG. *Singleton elective caesarean delivery episodes in NHS trusts (which had data for at least 50% of their deliveries in at least 7 years) from 1 April 2000 to 28 February 2009. Included if their HES record contained the code of ECS in any of the core operative procedure fields.* Exclusion criteria: Women who had an ECS < 34 weeks or a medical history. (e.g., preexisting and gestational diabetes, hypertensive disorders, preeclampsia, eclampsia, premature rupture of membranes, poly- and oligohydramnios, excessive or poor foetal growth, and placenta praevia).	ECS n, 118,456
Hutcheon 2015	Inclusion criteria: All ECS ≥ 37 WG for a primary indication of breech, repeat CS, or maternal request/vaginal birth after CS declined. Exclusion criteria: Pregnancies complicated by diabetes in pregnancy, antepartum haemorrhage, hypertension in pregnancy, or other comorbidities that might be an indication for an earlier delivery.	ECS n, 2021/2518 Maternal age, mean in years (SD): 33.7 (4.6)/34.1 (4.7) Nulliparous n (%): 352 (17.4)/486 (19.3) Prepregnancy BMI (kg/m2) n (%): Underweight (< 18.5), 80 (4.0)/93 (3.7) Normal weight (18.5–24.9), 872 (43.1)/1050 (41.7) Overweight (25–29.9), 254 (12.6)/352 (14.0) Obese (≥ 30), 98 (4.9)/137 (5.4) Missing, 717 (35.5)/886 (35.2) WG at delivery median in (days) (range), 271 (268–274)/273 (270–276) Birthweight mean in g (SD): 3420 (425)/3421 (426)
MacAllister 2019	Inclusion criteria: All ECS ≥ 37 WG (= absence of labour and no maternal or foetal indication for urgent delivery). Exclusion criteria: NR	ECS n, 19,780/40,875
Nicholl 2010	Inclusion criteria: All ECS ≥ 37 WG for ERCS, breech presentation, or maternal request. Exclusion criteria: Medical indication	NR
Nicoll 2004	Inclusion criteria: All ECS ≥37 WG (= timing of delivery could be postponed without any risk to the mother or foetus) Exclusion criteria: NR	ECS n, 292/327
Snowden 2016	Eligibility criteria: All ECS ≥ 37 WG (= without medical or obstetrical indication). Exclusion criteria: Women with a medical history or pregnancy-related complications (e.g., chronic hypertension, prepregnancy diabetes, and gestational diabetes mellitus).	ECS n, 12,204/7,697
Tanger 2010	Inclusion criteria: All ECS ≥37 WG Exclusion criteria: Women with a medical history or pregnancy-related complications (e.g., preeclampsia, maternal infection, (suspicion of) foetal distress, severe birth defects of the foetus, maternal gestational diabetes or diabetes mellitus).	ECS n, 324/486 Maternal age, mean in years (SD): 36.4 (4.7)/34.8 (4.2) Multiples n (%), 9 (3)/15 (3) Female n (%), 167 (50)/254 (51) Birthweight mean in g (SD): 3282 (470)/3386 (494) Apgar score after 5 min < 6; n (%), 0/0
Yamasato. 2014	Inclusion criteria: All ECS ≥ 37 WG. Exclusion criteria: Medical indication	NR

*CS* caesarean section, *ECS* elective caesarean section, *ERCS* elective repeat caesarean section, *WG* week of gestation

Implementation strategies differed very much between the studies. For a better comparability, we categorized the implementation strategies and split descriptions into “Category”. We determined following categories as soon as we had extracted the description of the implementation strategy: Regional quality reform, hospital internal quality reform, regional policy, local hospital policy, local hospital education, and publication of a guideline. For details, see Table 2. According to the intervention types listed above, we found an audit and feedback in Nicoll et al. [40], continuous quality improvement in Dunn et al. [38], and a multifaceted intervention in Nicholl et al. [41]. Moreover the local/regional policies are quality improvement projects without feedback and continuous learning [37, 42–44, 46]. The 2 studies assessing the impact of the publication of a guideline cannot be allocated to any of these strategies as there is no information if for example staff obtained written information about the guidelines or anything else [39, 45]. Additionally, we added descriptive information on the “rationale for implementation strategy” if available. We found two studies stated that the publication of a guideline [42, 43] was their rationale for the strategy and a systematic review and recommendations from the Registered Nurses’ Association of Ontario Toolkit was the rationale of the strategy in another study [38, 48]. No other study reported any rationale for their idea of the implementation strategy. Regarding the addressees and involvement of persons the Strategies differed. Obstetricians, midwifes, and neonatologists could be involved and it varied if for example the department chair was needed to give permission. In no study, the strategy was directed to the expectant mother. We also assessed the time of implementation and the follow-up time reported in the studies. Time of implementation ranged from 1998 in Nicoll et al. [40] to 2011 in Yamasato et al. and Allen et al. [43, 44] and follow-up was between 5 months in Nicholl et al. [41] and 6 years in Macallister et al. [39].

**Table 2:** Study characteristics

Study	Region, setting, data source, study design, funding	Intervention	Control	Outcomes
**Allen 2020**	South Carolina, USA All birthing hospitals National Vital Statistics data from the National Center for Health Statistics from 2009 to 2015 (Controlled) Before-after study* Funding: NR	Category: Regional policy (quality improvement) South Carolina Birth Outcomes Initiative (SCBOI) was at first (2011) voluntary for all South Carolina birthing hospitals, which implemented strategies such as patient and provider education. The state’s Medicaid director declared that if rates were not suitably reduced with the voluntary programme, he would institute a nonpayment policy for EED. 2013: “Hard-stop” Policy for a state (South Carolina). If an EED was not medically justified (defined as diabetes, hypertension, eclampsia, breech, and other pregnancy abnormalities, medical conditions present at the time of delivery like premature rupture of membranes, prolonged labour, foetal distress; according to Joint Commission’s conditions possibly justifying delivery < 39 weeks), the hospital would attempt to “hard stop” the procedure from being scheduled. Medicaid and Blue Cross Blue Shield (covering 85% of births in South Carolina) both followed the policy. Rationale for implementation strategy: South Carolina having the 4th highest EED rate in the country Implementation of intervention: Voluntary programme September 2011, “hard-stop” January 2013 Period after intervention: September 2011 to December 2012 voluntary programme, “hard-stop” January 2013–September 2015	No policy implemented Period before intervention: 2009–September 2011	Primary: EED rate at term < 39 WG Supplementary analysis: ECS rate at term < 39 WG
**Dunn 2013**	Eastern Ontario, Canada 10 hospitals of a local health integration network (1 level 3, 3 level 2, 6 level 1) Database BORN Ontario (Birth Record Database 2009–2010 and 2010–2011) Before-after study Funding: NR	Category: Regional quality reform (continuous quality improvement) Incentive-based quality improvement project setting the rate of ERCS at term in low-risk women performed < 39 WG to 30% as a quality indicator - Letter describing the project - Site specific rates - Custom query report instructions for data retrieval - Chart audit tool to review cases - Knowledge-to-action plan - BIS birth record definitions - Knowledge-to-action evidence summary - 6 months follow-up call Rationale for implementation strategy: Chaillet et al. and recommendations from the Registered Nurses’ Association of Ontario Toolkit [36, 48] Implementation of intervention: 31 March 2010 Period after intervention: 01 April 2010–31 March 2011	No quality reform implemented Period before intervention: 01 April 2009–31 March 2010	Primary: ERCS rate at term < 39 WG Adjustment: No adjustment
**Gurol-Urganci 2014**	England 63 NHS trust Database (routinely collected HES database captures patient demographics and clinical information for all admissions to English NHS trusts) Interrupted time series Funding: NR	Category: Publication of a guideline 2004 NICE Guideline: caesarean section. Recommendation: planned CS should not routinely be carried out before 39 weeks [11] Rationale for implementation strategy: NA Implementation of intervention: April 2004 Period after intervention: April 2004–28 February 2009	No guideline published Period before intervention: 01 April 2000–01 April 2004	Primary: ECS rate at term ≥ 39 WG Adjustment: No adjustment
**Hutcheon 2015**	Vancouver, Canada British Columbia Women’s Hospital Tertiary care teaching hospital Hospital database, which contains linked clinical, administrative, and operating room databases. These include the BC Perinatal Database Registry, the Canadian Institute for Health Information’s Discharge Abstract Database, and the hospital surgery scheduling records (ORSOS) Interrupted time series Funding: - One author holds New Investigator awards from the Canadian Institutes of Health Research and the Michael Smith Foundation for Health Research. - Two authors hold Chercheur- Boursier awards from the Fonds de Recherche en Santé Quebec.	Category: Local hospital policy (quality improvement) Limitation for low-risk planned CS < 39 WG at the level of the operating room booking clerk. Operating room booking clerk required confirmation of WG of at least 39 + 0 based on the last menstrual period, revised with early ultrasound using the algorithm from the Society of Obstetricians and Gynecologists of Canada, prior to booking the surgery. Rationale for implementation strategy: NR Implementation of intervention: 01 April 2008 Period after intervention: 01 April 2008–31 March 2012	No policy implemented (the timing of a planned CS was at the discretion of the attending physician) Period before intervention: 01 April 2005–31 March 2008	Primary: CS rate at term < 39 WG Adjustment: maternal age, prepregnancy body mass index, and number of previous CS
**Macallister 2019**	Western Australia Database MNS, NETS WA database and neonatal unit admission records. The MNS receives notifications on all midwifery attended births in WA. The NETS WA database contains information on all aspects of the retrieval process Before-after study Funding: NR	Category: Publication of a guideline 2006 RANZCOG guideline: Timing of elective caesarean section at term Recommendation: It is recommended that elective caesarean section in women without additional risks should be carried out at approximately 39 WG [13] Rationale for strategy: NA Implementation of intervention: November 2006 Period after intervention: 01 January 2008–31 December 2014	No guideline published Period before intervention: 01 January 2003–31 December 2006	Primary: CS rate at term < 39 WG Adjustment: No adjustment
**Nicholl 2010**	New-South-Wales, Australia Tertiary referral hospital Local database Before-after study Funding: NR	Category: Local hospital education (multifaceted intervention) Developed by: Obstetric consultant, delivery suite midwifery manager, clinical research midwife, delivery suite staff, quality improvement advisor, maternity data analyst Intervention: Pre-emptive education of midwifery/ obstetric staff, evidence folders in key clinical areas, background data/objectives discussed at clinical meetings focusing antenatal clinic/delivery suite. Process change on dating/booking system: - Indications for CS mandatory at booking, as WG - Delivery suite staff refer on to Clinical Director CS booking without clinical indication for delivery < 39 WG. Criteria: maternal or foetal condition that would benefit from early delivery (local clinical database) Rationale for implementation strategy: NR Implementation of intervention: March 2007 to August 2007 Period after intervention: NR	Booking system: direct referral from clinicians in outpatients department, wards/private consulting rooms to delivery suite staff, only basic details required to complete the booking. No screening of indication for the procedure in place. Period before intervention: 2005–2006	Primary: CS rate at term < 39 WG NICU admission Adjustment: No adjustment
**Nicoll 2004**	Glasgow, Scotland Royal Maternity Hospital, Glasgow Registry and operating theatre books. (Labour ward register of births) Before-after study Funding: NR	Category: Local hospital quality reform (audit and feedback) Recommendation to delay ECS ≥ 39 WG without obstetric indication for early-term delivery. An audit was performed before and after the intervention. The results of the first audit cycle were presented to obstetric and paediatric staff. Afterwards recommendation was given. WG was measured with last menstrual period and ultrasound in week 20. Rationale for implementation strategy: NR Implementation of intervention: 1 January 1998 Period after intervention: June 1999–June 2000	No quality reform implemented Period before intervention: Cycle I: October 1996–October 1997	Primary: CS rate at term < 39 WG Secondary: NICU admission Adjustment: No adjustment
**Snowden 2016**	Oregon, USA 49 hospitals providing maternity care Database (vital statistics data provided by the Oregon Center for Health Statistics) Before-after study Funding: Supported by the Health Resources and Services Administration (HRSA) of the US Department of Health and Human Services (HHS) under Policy R40 Award (number R40 MC268090201). One author is supported by the Eunice Kennedy Shriver National Institute of Child Health and Human Development (grant number R00 HD079658-03).	Category: Regional policy (quality improvement) “Hard-stop” policy for a state (Oregon). The policy limited early-term deliveries by requiring review and approval for any delivery without documented indication (gestational hypertension, preeclampsia, eclampsia, foetal growth restriction) < 39 WG Rationale for implementation strategy: NR Implementation of intervention: 2011 Period after intervention: 2012–2013 [2011 excluded, because of unexposed time periods of intervention]	No policy implemented Period before intervention: 2008–2010	Primary: CS rate at term < 39WG Secondary: NICU admission Adjustment: multivariable logistic regression for maternal race/ethnicity, parity, insurance status, prenatal care, maternal age and education, certified nurse-midwife attendant
**Tanger 2010**	Amsterdam, the Netherlands VU University Medical Center Database and registry; National Pediatrician Registration Database, the 2nd line (LVR2) and operating registrations (OPERA) selected on the Primary Sector Code CS. Then, both data sets were combined to one complete database Before-after study Funding: None	Category: Local hospital policy (quality improvement) ECS will be planned ≥ 39 WG in the absence of comorbidities (preeclampsia, maternal infection, (suspicion of) foetal distress, severe birth defects of the foetus, maternal gestational diabetes, or diabetes mellitus). According to the protocol, every ECS indication was resolved in the weekly meeting of paediatricians and obstetrics. WG was measured with ultrasound in the first trimester. Rationale for implementation strategy: NICE Guideline CG13 and ACOG Committee Opinion No. 394 [11, 49] Implementation of intervention: NR Period after intervention: January 2003–December 2007	No policy implemented Period before intervention: 1994–1998	Primary: CS rate at term ≥ 39 WG Adjustment: No adjustment
**Yamasato 2014**	Honululu, Hawaii Kapi’olani Medical Center for Women and Children Database (outcomes obtained from data fields in maternal and neonatal charts) Before-after study Funding: Hawaii Pacific Health Research Institute	Category: Local hospital policy (quality improvement) Any delivery induction required the patient to be ≥ 39 WG and by ACOG dating criteria or have a medical condition (according ACOG and the Joint Commission National Quality Measures for Perinatal Care) justifying induction. In the absence of a medical indication for induction, a minimum Bishop score of 6 is required. Inductions not meeting criteria were not to be scheduled without approval by the Department Chair. WG was measured according to the ACOG practice bulletin No. 107 [50] Rationale for implementation strategy: ACOG practice bulletin no. 107 [50] Implementation of intervention. 2011 Period after intervention: 2010–31 March 2012	No policy implemented Period before intervention: 01 June 2010–2011	Primary: Induction rates at term ≤ 39 WG Adjustment: Multivariable logistic regression on maternal characteristics

Category: Type of intervention (regional quality reform, hospital internal quality reform, regional policy, local hospital policy, local hospital education, publication of a guideline)

*Authors controlled with number from other states without any strategy to reduce early elective induction. However, in the subgroup of early elective CS, no control was reported

*ACOG* The American Congress of Obstetricians and Gynecologists, *BIS* BORN Information System, *BORN* Better Outcomes Registry & Network, *CS* caesarean section, *ECS* elective caesarean section, *EED* early elective delivery, *ERCS* elective repeat caesarean section, *HES* hospital episode statistics, *MNS* midwives notification system, *NA* not applicable, *NETS WA* Newborn Emergency Transport Service Western Australia, *NHS* National Health Services, *NICE* The National Institute for Health and Care Excellence, *NICU* neonatal intensive care unit, *NR* not reported, *RANZCOG* Royal Australian and New Zealand College of Obstetricians and Gynecologists, *WA* Western Australia, *WG* week of gestation

All comparators were the time before implementation of the strategy.

### Risk of bias within studies

We assessed risk of bias with the ROBINS-I tool. Consistently throughout all studies, confounding was the main issue and we assumed moderate risk of bias in 4 studies [37, 44–46] while critical or serious in the other 6, see Table 3. Those, which were rated “critical”, did not approach any adjustments. Yamasato et al. controlled for confounding but did not report adjusted results of our primary outcome [43]. Main confounding factors we identified were maternal age and maternal and neonatal comorbidities. However, we also saw confounding regarding study setting and health staff, most importantly that they were not blinded. Only four studies reported how they measured WG [40, 42, 43, 46]. We did not identify any risk of bias for selection of participants or classification of intervention as all included clinics/all health stuff received the intervention and all studies classified the groups before and after intervention clearly. We assessed 2 studies with serious risk of bias [38, 39], 3 studies did not report on adhering to the intervention [40–42] and we rated the others with low or moderate risk of bias depending on the potential confounding through co-interventions. We rated serious risk of bias due to missing data in one study [45]. One study was rated with moderate [43] and one with low [37] risk of bias due to missing data; all others were rated with no information. We rated Snowden et al. [37] with serious risk of bias in measurements of outcomes as the authors stated they assumed systematic errors in documentation due to the national attention through the implemented hard-stop policy. We rated all other studies with moderate or low risk of bias. Moreover, for the assessment of selective reporting of results, we rated all studies with low, except two [44, 46] with moderate risk of bias. Concerning overall risk of bias we rated only two studies [44, 46] as having moderate risk of bias, the rest had an either serious or critical overall risk of bias. The detailed ratings to each bias domain can be found in appendix C.

**Table 3:** Risk of bias assessment with ROBINS-I

Study	Outcome	1	2	3	4	5	6	7	Overall
Allen 2020	CS rate	M	L	L	L	NI	L	M	M
Dunn 2013	CS rate	C	L	L	S	NI	L	L	C
Gurol-Urganci 2014	CS rate	M	M	L	L	S	L	L	S
Hutcheon 2015	CS rate	M	L	L	L	NI	L	M	M
MacAllister 2019	CS rate	C	L	L	S	NI	L	L	C
Nicholl 2010	CS rate, NICU admission	C	L	L	NI	NI	M	L	C
Nicoll 2004	CS rate, NICU admission	C	L	L	NI	NI	M	L	C
Snowden 2016	CS rate, NICU admission	M	L	L	M	L	S	L	S
Tanger 2010	CS rate	C	L	L	NI	NI	L	L	C
Yamasato 2015	CS rate	S	L	L	M	M	L	L	S

Risk of bias options are L, low; M, moderate; S, serious; C, critical; and NI, no information

*1* bias due to confounding, *2* bias in selection of participants into the study, *3* bias in classification of interventions, *4* bias due to deviations from intended interventions, *5* bias due to missing data, *6* bias in measurement of outcomes, *7* bias in selection of the reported result, *CS* caesarean section assessment according to ROBINS-I tool

The seven bias domains are individually assessed for each study

### Risk of bias across studies

The assessment resulted in serious or critical risk of bias for the majority of studies. The 2 interrupted time series studies were rated as having a serious and moderate risk of bias [45, 46]. By contacting the study authors, we received only one funding application from Hutcheon et al. [46] showing differences in planned outcome assessment compared to the publication. The authors explained that with (non-)availability of data. We used this for the assessment of selective reporting, as no study protocol was available.

### Results of individual studies

**Fig. 2:**
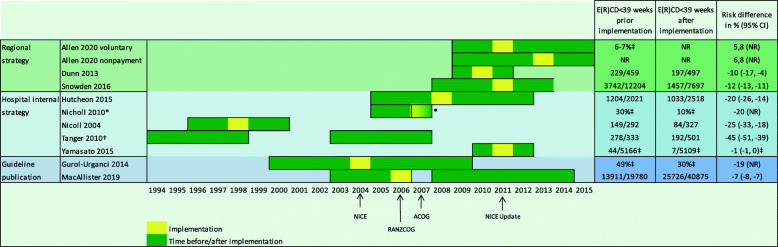
Results for decrease of elective CS < 39 WG in individual studies. ACOG, American College of Obstetricians and Gynecologists; CI, confidence interval; E(R)CD, elective (repeat) caesarean d; NICE, National Institute of Health and Care Excellence; RANZCOG, Royal Australian and New Zealand College of Obstetricians and Gynaecologists. Asterisk indicates transition period, full implementation unclear. Dagger indicates implementation period unclear. Double dagger indicates no absolute numbers of elective CS reported

Strategies postponing elective CS to ≥ 39 WG were effective in all studies. All studies, which reported CI, showed statistical significance. Hutcheon et al., one of the two studies rated with moderate risk of bias showed a risk difference of − 20% (CI 95% − 26%, − 14%). They followed a local hospital policy. They were the only authors reporting adjusted risk differences for maternal age, prepregnancy body mass index, and number of previous CS. The adjusted risk difference showed the same values as the unadjusted [46]. Only Snowden et al., following a regional policy, reported adjusted values as well. They report an unadjusted risk difference for elective CS < 39 + (0–6) WG of − 12% (CI 95% − 13%, − 11%) after implementation and an adjusted odds ratio of 0.6 (CI 95% 0.58, 0.64). Adjustment was for maternal race/ethnicity, parity, insurance status, prenatal care, age, education, and certified nurse-midwife attendant [37]. Individual study results for the rate of elective CS < 39 + (0–6) WG are displayed in Fig. 2. Three studies reported NICU admission rates. Nicoll et al. reported 11 prevented cases after implementation (CI 95% 2, 24) [40]. Snowden et al. reported an adjusted odds ratio of 1.03 (CI 95% 0.97, 1.10) post implementation but the denominator was all births (not only elective CS) [37]. Nicholl et al. showed a reduction of NICU admission for neonates with an elective CS < 39 + (0–6) after implementation of the intervention to no admission but did not report CI or significance [41].

### Synthesis of results

Figure 2 shows the results of the included studies following the timeframe observed from 1994 to 2015. We show the time when the implementation of the intervention has taken place and also when a guideline was published. Some studies reported an actual date of the implementation, some indicated a year or month. We showed all strategies on the timeline as an interval of one year, e.g., implementation of intervention on 1 January 1998 is depicted as 1998–1999. Nicholl et al. did not report the period after the intervention. It is unclear when the strategy was fully implemented and how long follow-up took [41]. Tanger et al. only specified the pre- and post-period but did not report when the intervention was implemented in between these timepoints [42]. We present numbers of elective CS < 39 + (0–6) WG and numbers of elective CS prior to the implementation of the intervention and after the implementation. One study reports an adjusted OR for elective CS < 39 + (0–6) WG with time prior to the implementation as the reference [37]. As the only authors, Hutcheon et al. reported the risk difference adjusted to various confounders [46].

As stated above, all studies showed a reduction of elective CS < 39 + (0–6) WG after implementation of the intervention. The biggest difference can be seen in Tanger et al. with a risk difference of − 45% (CI 95% − 51%, − 39%) but also the longest observation period of 13 years [42]. There were 4 studies which did not report the number of elective CS < 39 + (0–6) WG or the total number of elective CS [41, 43–45]. Studies, which used a regional implementation strategy reported a risk difference of −5.8 to −6.8% (CI not reported), − 10% (CI 95% − 17%, − 4%) and − 12% (CI 95% − 13%, − 11%) [37, 38, 44]. Two studies assessed the change after the publication of a guideline (NICE in Gurol-Urganci et al. and RANZCOG in Macallister et al.). Gurol-Urganci et al. showed a risk difference of 19% (CI 95% not reported) and Macalister et al. 7% (95% CI − 8, − 7). These studies observed the highest number of elective CS in various hospitals with n = 118,456 and n = 60,655, respectively [39, 45]. The other studies implemented various local hospital implementation strategies and results ranged from − 20 (CI 95% − 26%, − 14%) [46] to − 45% (CI 95% − 51%, − 39%) [40–43].

## Discussion

### Summary of evidence

We found that overall all studies assessing implementation strategies to shift elective CS < 39 + (0–6) WG to ≥ 39 + (0–6) WG showed a successful reduction of elective CS < 39 + (0–6) WG. Except for one study, risk of bias was serious or critical in all studies. We could see a small difference regarding the scope of the implementation strategy, it seems that local hospital strategies may lead to a greater success in decreasing elective CS < 39 + (0–6) WG compared to regional strategies or the publication of a guideline. However, we could not see any differences in how the strategy was used and if specific aspects of the strategies, e.g., who was involved, lead to better results. There is a hint that strategies in single hospitals might be more successful than in a regional hospital group. We saw an audit and feedback in Nicoll et al. [40], continuous quality improvement in Dunn et al. [38] and a multifaceted intervention in Nicholl et al. [41]. We rated the local/regional policies assessed in 5 studies as quality improvement projects (differing from continuous quality improvement with a feedback circle) [37, 42–44, 46]. We could not see a difference in effectiveness according to the intervention type [36].

### Limitations

We identified serious or critical risk of bias in most included studies due to the main issues of confounding and some of missing data and lack of blinding. There are various aspects of confounding that only 2 studies adjust for, like maternal risks, age, race, or body mass index. However, there is also confounding in most studies regarding the setting or the health stuff involved. For example, Dunn et al. discuss limited access to operating rooms or limited paediatricians at certain times [38]. Moreover, Nicoll et al. say that senior obstetrics health stuff had fixed sessions at labour ward when they were available for CS and may have summon their patients in these times [40]. Although it was not clear in most studies if and what kind of co-interventions may have influenced the outcome, especially in those that were looking at a longer time span. For example, Snowden et al. state that there are known changes in health care systems and organization in the state during the analysed time. In post-period, the state transformed its medicaid programme, which affected the organization of health care delivery for publicly insured pregnant women [37]. Especially coding seems to have a large impact. On the one hand, the implementation strategies put a focus on coding; on the other hand, a change of coding may appeared through that focus. For example, Gurol-Urganci et al. state that incomplete coding of the diagnosis and indication for elective CS may have led to an underestimation of the proportion of elective CS ≥ 39 + (0–6) WG [45]. While Macallister et al. say that there is a reduced diagnosis of cephalo-pelvic disproportion, but an increase in elective CS for medical reasons before and after the guideline publication which might be due to an (un-)conscious change of coding [39]. Not all of the included studies reported the method of estimation of gestational age, which is one of the main aspects to know for choosing the right time point. Who measures gestational age and how it is measured should be part of any implementation strategy and should be reported. There was no information on implementation fidelity reported. However, Nicoll et al. and Dunn et al. used an audit as part of their implementation strategy; it is possible that an audit enhance implementation fidelity [38, 40].

Our review has a few limitations. Our main limitation is that we were very inclusive by choosing the studies, which resulted in very high heterogeneity and no meta-analysis was possible. We included studies considering elective CS with and without medical indication, other elective birth modes, and one without providing additional analysis [43]. We tried to get in contact with the authors of the studies but without success. Gurol-Urganci et al. reported patients from 34 + 0 WG on, which is not term birth yet. They reported those from 37th separately but still it is not clear if all births are dated to 37 + 0–6 WG and older. And Nicholl et al. did not report any follow-up; they only measured the time during the implementation of the strategy to shift the timing of elective CS. We did not specify to consider studies assessing multiple hospitals only. One can assume that hospitals with a very high rate on elective CS < 39 + (0–6) WG or without any structured guideline on planning elective CS would rather have conducted such implementation projects. In this case, there might be a larger effect in the reduction. Studies assessing data of only a single hospital need to be considered more carefully. Even though we found 2 studies only reporting the impact of the guideline publication, we could not differentiate how the publications of guidelines may have influenced the results on other implementation strategies. Even a general focus on timing of elective CS through the guidelines or other research could have affected the results. Considering that we included studies with various time spans, from the 90s to 2015, general changes in obstetrical practice and education may have impacted the results.

One benefit of our review is that we created a comprehensive overview of various strategies used and assessed to postpone elective CS to late term. For planning a similar implementation, our review gives some useful hints. It is essential to consider barriers and limitations specific to the medical specialty. Although, barriers and limitations of the specific context must be known or evaluated if possible [51]. Most of our included studies identified barriers and evaluated the influence on success. Depending on the structure, hierarchy, and status of guideline implementation, an interested hospital (or group of hospitals) could follow one of the comprehensively described strategies presented here. One may find a similar clinical setting as presented in the studies and a strategy fitting in their individual setting.

### Agreement with other studies and transferability of results

A study on strategies postponing induction of birth to late term showed that hard-stop policies (= not allowed to perform early-term induction without medical indication) compared to education and policies left up the physician are the most successful [52]. There is already research about implementation strategies lowering the CS rate in general. The meta-analysis by Chaillet et al., including 10 studies on different implementation strategies to reduce CS, found that interventions involving all obstetrical staff in analysing and modifying their practice can safely lower the CS rate [36]. Obstetrical staff needs to be involved to identify barriers on the one hand and receive feedback after implementation of the strategy on the other hand, according to the authors. We found that various strategies on shifting elective CS < 39 + (0–6) to ≥ 39 + (0–6) WG are effective and resulted in a reduction of elective CS < 39 + (0–6) WG. We only found little data on the effect on NICU admission rates, but they seem to decrease little. It remains unclear which strategy is more effective or which aspects of a strategy should be considered in future implementation strategies. There is a little hint that strategies on an individual hospital level have the largest effect. However, it is not clear if the effect may result from confounding through a higher need in general structured planning of elective CS in these hospitals. In addition, confounding through stricter coding may have a higher influence in these hospitals. In general, methodological quality of the studies was low. Moreover, it remains unclear how much impact the publications of various guidelines, stating elective CS to be performed in ≥ 39 + (0–6) WG, have or how general changes in attitudes, education and research affected the results. The first publication of the recommendation on timing by NICE was in 2004. Except Nicoll et al., our included studies have been obtained afterwards. Maybe a general reduction of elective CS < 39 + (0–6) WG have taken place since then and the effect seen in the studies might be a result of this. However, there is no actual data comparing WG in elective CS nowadays compared to 10 or 15 years ago. On the other hand, even though the recommendation is known for quite a long time now, it is possible that it is not implemented for various reasons. There might be non-awareness of the guidance, hierarchical, and antiquated structures or rural areas where spontaneous labour resulting in an emergency CS may be a danger because of longer travel times to the next obstetrical clinic. The recommendation could even be implemented and recommended to the expectant mother, but the reality of conducting elective CS can still differ. On the one hand, the wish of the expectant mother is included which may result in an early-term date (e.g., because of anxiety or discomfort in late pregnancy). On the other hand, the supervising gynaecologist may not be aware of the recommendation and advising early term. It is already known that physicians in an ambulatory setting adhere less to guidelines compared to physicians in a hospital [53]. An analysis of health insurance data would show if there is an effect only by publication of the guidance, comparing the last 15 years.

## Conclusions

There are substantially unexplained variations in obstetrical practice, especially when it comes to induction of birth and planning of elective CS. Numerous guidelines give recommendations on the timing of elective CS aiming to increase quality in health care, but physicians and other obstetrician staff face difficulties in rapidly integrating evidence into their practice. For a successful knowledge transfer and integration, it is essential to promote strategies that reach those involved sustainably [54]. In general, any implementation strategy to shift elective CS < 39 + (0–6) to ≥ 39 + (0–6) WG should be flexible when it comes to maternal and neonatal comorbidities or characteristics as age or BMI. Moreover, all involved obstetric staff should be included and settings like operating rooms and schedules must be prepared (e.g., availability) and constructed for the change. Our review may give details to those who are planning an implementation strategy for the reduction of elective CS < 39 + (0–6) WG by providing summaries of studies which have shown a successful reduction. The evidence suggests implementing shifting elective CS from early to late term rather at a single hospital base considering the specific barriers and facilitators.

## Supplementary Information

**Additional file 1: Appendix A.** Search strategies. **Appendix B.** Included and excluded studies **Appendix C.** - Risk of bias assessment with ROBINS-I.

## Data Availability

The datasets generated and analysed during the current study will be available from the corresponding author on reasonable request.

## Abbreviations

ACOG: American College of Obstetricians and Gynecologists
CI: Confidence interval
CS: Caesarean section
DGGG: German Society of Gynecologists and Obstetricians
NICE: National Institute for Health and Care Excellence
NICU: Neonatal intensive care unit
RANZCOG: Royal Australian and New Zealand College of Obstetricians and Gynaecologists
RCOG: Royal College for Obstetricians and Gynecologists
RCT: Randomized controlled trial
VBAC: Vaginal birth after caesarean section
WHO: World Health Organization
WG: Week of gestation
