# Supplementary material for: A systematic review on the effectiveness of implementation strategies to postpone elective caesarean sections to ≥ 39 + (0–6) weeks of gestation
Source: Syst Rev. 2021 Jun 14;10:176. doi: 10.1186/s13643-021-01718-1 (PMC8201675; doi:10.1186/s13643-021-01718-1)
Supplement: Supplementary file 1 — Additional file 1: Appendix A. Search strategies. Appendix B. Included and excluded studies Appendix C. - Risk of bias assessment with ROBINS-I. [file 13643_2021_1718_MOESM1_ESM.docx]

Appendix A: Search strategies

Search strategy for MEDLINE:

| (((Cesarean[tiab] OR Caesarean[tiab] OR Cesarian[tiab] OR Caesarian[tiab] OR "Cesarean Section"[Mesh] OR C-Section[tiab] OR “C Section”[tiab] OR “C Sections”[tiab]) AND (Timing[tiab] OR late[tiab] OR prior[tiab] OR delayed[tiab] OR time[tiab] OR week*[tiab]) AND Elective[tiab]) AND (guidance OR guideline* OR information* OR education OR audit* OR policy OR policies OR strategy* OR pamphlet* OR leaflet*)) |
| --- |

Search strategy Embase

| ((('cesarean section'/exp OR (c$esar?an OR “c-section*”):ti,ab,kw) AND ((timing OR late OR prior OR delayed OR time OR week?):ti,ab,kw) AND elective:ti,ab,kw) AND ('guidance'/exp OR 'practice guideline'/exp OR 'guideline'/exp OR 'information'/exp OR 'education'/exp OR 'policy'/exp OR 'publication'/exp OR (guidance OR guideline$ OR information$ OR education OR audit$ OR polic* OR strateg* OR pamphlet$ OR leaflet$):ti,ab,kw)) AND ([embase]/lim) AND (embase NOT (embase AND medline)) NOT (('comment' OR 'letter' OR 'editorial'):it) AND ([1-1-2000]/sd NOT [16-11-2019]/sd) |
| --- |

Limited to EMBASE and Articles, Articles + press, Reviews

Search strategy Cinahl (via EBSCO)

| S13 | S12 AND S11 | Limiters - Published Date: 20000101-20191231  Expanders - Apply equivalent subjects  Search modes - Boolean/Phrase | Interface - EBSCOhost Research Databases  Search Screen - Advanced Search  Database - CINAHL | 240 |
| --- | --- | --- | --- | --- |
| S12 | S3 AND S4 AND S5 | Expanders - Apply equivalent subjects  Search modes - Boolean/Phrase | Interface - EBSCOhost Research Databases  Search Screen - Advanced Search  Database - CINAHL | 1,076 |
| S11 | S6 OR S7 OR S8 OR S9 OR S10 | Expanders - Apply equivalent subjects  Search modes - Boolean/Phrase | Interface - EBSCOhost Research Databases  Search Screen - Advanced Search  Database - CINAHL | 949,592 |
| S10 | TI ( guidance OR guideline* OR information* OR education OR audit* OR polic* OR strateg* OR pamphlet* OR leaflet* ) OR AB ( guidance OR guideline* OR information* OR education OR audit* OR polic* OR strateg* OR pamphlet* OR leaflet* ) | Expanders - Apply equivalent subjects  Search modes - Boolean/Phrase | Interface - EBSCOhost Research Databases  Search Screen - Advanced Search  Database - CINAHL | 938,658 |
| S9 | (MM "Pamphlets") | Expanders - Apply equivalent subjects  Search modes - Boolean/Phrase | Interface - EBSCOhost Research Databases  Search Screen - Advanced Search  Database - CINAHL | 1,121 |
| S8 | (MM "Audit") | Expanders - Apply equivalent subjects  Search modes - Boolean/Phrase | Interface - EBSCOhost Research Databases  Search Screen - Advanced Search  Database - CINAHL | 4,085 |
| S7 | (MM "Education") | Expanders - Apply equivalent subjects  Search modes - Boolean/Phrase | Interface - EBSCOhost Research Databases  Search Screen - Advanced Search  Database - CINAHL | 3,678 |
| S6 | (MM "Practice Guidelines") | Expanders - Apply equivalent subjects  Search modes - Boolean/Phrase | Interface - EBSCOhost Research Databases  Search Screen - Advanced Search  Database - CINAHL | 26,508 |
| S5 | TI elective OR AB elective | Expanders - Apply equivalent subjects  Search modes - Boolean/Phrase | Interface - EBSCOhost Research Databases  Search Screen - Advanced Search  Database - CINAHL | 17,842 |
| S4 | TI ( timing OR late OR prior OR delayed OR time OR week* ) OR AB ( timing OR late OR prior OR delayed OR time OR week* ) | Expanders - Apply equivalent subjects  Search modes - Boolean/Phrase | Interface - EBSCOhost Research Databases  Search Screen - Advanced Search  Database - CINAHL | 843,853 |
| S3 | S1 OR S2 | Expanders - Apply equivalent subjects  Search modes - Boolean/Phrase | Interface - EBSCOhost Research Databases  Search Screen - Advanced Search  Database - CINAHL | 23,965 |
| S2 | TI ( c#esar?an OR “C-section*” ) OR AB ( c#esar?an OR “C-section*” ) | Expanders - Apply equivalent subjects  Search modes - Boolean/Phrase | Interface - EBSCOhost Research Databases  Search Screen - Advanced Search  Database - CINAHL | 18,508 |
| S1 | (MH "Cesarean Section+") | Expanders - Apply equivalent subjects  Search modes - Boolean/Phrase | Interface - EBSCOhost Research Databases  Search Screen - Advanced Search  Database - CINAHL | 16,400 |

Search strategy CENTRAL

| ID Search Hits  #1 MeSH descriptor: [Cesarean Section] explode all trees 2956  #2 (Cesarean OR Caesarean OR Cesarian OR Caesarian OR C-Section OR “C Section” OR “C Sections”):ti,ab,kw 12106  #3 #1 OR #2 12106  #4 (Timing OR late OR prior OR delayed OR time OR week?):ti,ab,kw 672646  #5 (elective):ti,ab,kw 26179  #6 #3 AND #4 AND #5 1549  #7 MeSH descriptor: [Education] explode all trees 30885  #8 MeSH descriptor: [Policy] explode all trees 731  #9 MeSH descriptor: [Pamphlets] explode all trees 852  #10 (guidance OR guideline? OR information? OR education OR audit? OR polic* OR strateg* OR pamphlet? OR leaflet?):ti,ab,kw 216803  #11 #7 OR #8 OR #9 OR #10 223105  #12 #6 AND #11 with Cochrane Library publication date Between Jan 2000 and Dec 2019 185 |
| --- |

Appendix B: Included and excluded studies

Table 1: Included studies

| Allen, L. and D. Grossman (2020). "The impact of voluntary and nonpayment policies in reducing early-term elective deliveries among privately insured and Medicaid enrollees." Health Services Research 55(1): 63-70. | |
| --- | --- |
| Dunn S, Sprague AE, Fell DB, Dy J, Harrold J, Lamontagne B, et al. The Use of a Quality Indicator to Reduce Elective Repeat Caesarean Section for Low-Risk Women Before 39 Weeks' Gestation: The Eastern Ontario Experience. Journal of Obstetrics and Gynaecology Canada. 2013;35(4):306-16. |  |
| Gurol-Urganci I, Cromwell DA, Edozien LC, Onwere C, Mahmood TA, van der Meulen JH. The timing of elective caesarean delivery between 2000 and 2009 in England. BMC Pregnancy & Childbirth. 2011;11(1):43-. |  |
| Hutcheon JA, Strumpf EC, Harper S, Giesbrecht E. Maternal and neonatal outcomes after implementation of a hospital policy to limit low-risk planned caesarean deliveries before 39 weeks of gestation: an interrupted time-series analysis. Bjog. 2015;122(9):1200-6. |  |
| Macallister KJ, Tho LW, Epee-Bekima M, Resnick S, Davis JW. Impact of elective caesarean section on neonatal retrieval in Western Australia during a 12-year period. J Perinatol. 2019;39(1):34-8. |  |
| Nicoll AE, Black C, Powls A, Mackenzie F. An audit of neonatal respiratory morbidity following elective caesarean section at term. Scottish medical journal. 2004;49(1):22-5. |  |
| Nicholl MC, Cattell MA. Getting evidence into obstetric practice: appropriate timing of elective caesarean section. Australian health review : a publication of the Australian Hospital Association. 2010;34(1):90-2. |  |
| Snowden JM, Muoto I, Darney BG, Quigley B, Tomlinson MW, Neilson D, et al. Oregon's Hard-Stop Policy Limiting Elective Early-Term Deliveries: Association With Obstetric Procedure Use and Health Outcomes. Obstetrics & Gynecology. 2016;128(6):1389-96. |  |
| Tanger HL, van den Berg A, Bolte AC, van Elburg RM. [Less neonatal morbidity with elective caesarean sections at term: local guideline for elective caesarean section is effective]. Nederlands tijdschrift voor geneeskunde. 2010;154:A1201. |  |
| Yamasato K, Bartholomew M, Durbin M, Kimata C, Kaneshiro B. Induction Rates and Delivery Outcomes After a Policy Limiting Elective Inductions. Maternal & Child Health Journal. 2015;19(5):1115-20. |  |

Table 2: excluded studies (with reason)

| Alderdice, F., et al., Timing of elective caesarean section at term: barriers to guidelines in practice. J Matern Fetal Neonatal Med, 2005. 17(2): p. 137-8. | Intervention |
| --- | --- |
| Ashton, D.M., Elective delivery at less than 39 weeks. Curr Opin Obstet Gynecol, 2010. 22(6): p. 506-10. | Study type |
| Berrien, K., et al., The perinatal quality collaborative of North Carolina's 39 weeks project: a quality improvement program to decrease elective deliveries before 39 weeks of gestation. N C Med J, 2014. 75(3): p. 169-76. | Study type |
| Bowman, D.S. and K. LiVolsi, Beyond 39 Weeks: Sustainability in Decreasing Cesarean Rates in Elective Inductions. JOGNN: Journal of Obstetric, Gynecologic & Neonatal Nursing, 2014. 43(Supp 1): p. S59-S59. | Population |
| Chescheir, N. and M.K. Menard, Scheduled Deliveries: Avoiding Iatrogenic Prematurity. American Journal of Perinatology, 2012. 29(1): p. 27-33. | Study type |
| Chesis, N., A Quality Improvement Project to Reduce the Incidence of Nonmedically Indicated Elective Deliveries Before 39 Weeks...Proceedings of the 2015 AWHONN Convention. JOGNN: Journal of Obstetric, Gynecologic & Neonatal Nursing, 2015. 44: p. S49-50. | Study type |
| Dahlen, H.M., et al., Texas Medicaid Payment Reform: Fewer Early Elective Deliveries And Increased Gestational Age And Birthweight. Health Aff (Millwood), 2017. 36(3): p. 460-467. | Population |
| Dixon, C.L., et al., Effect of a "Hard Stop" on Elective Labor Inductions and Cesarean Delivery Rate. J Reprod Med, 2016. 61(9-10): p. 411-415. | Population |
| Ehrenthal, D.B., et al., Neonatal outcomes after implementation of guidelines limiting elective delivery before 39 weeks of gestation. Obstet Gynecol, 2011. 118(5): p. 1047-55. | Population |
| Fisch, J.M., et al., Labor induction process improvement: a patient quality-of-care initiative. Obstet Gynecol, 2009. 113(4): p. 797-803. | Population |
| Heinrich, D., R.I. Vogel, and K.B. Kozhimannil, Early elective delivery and vaginal birth after cesarean in rural US maternity hospitals. Rural Remote Health, 2016. 16(4): p. 3956. | Population |
| Kennedy, E.B., et al., NICU Admissions After a Policy to Eliminate Elective Early Term Deliveries Before 39 Weeks' Gestation. Hosp Pediatr, 2018. 8(11): p. 686-692. | Population |
| Laye, M.R. and E.H. Dellinger, Timing of scheduled cesarean delivery in patients on a teaching versus private service: adherence to American College of Obstetricians and Gynecologists guidelines and neonatal outcomes. Am J Obstet Gynecol, 2006. 195(2): p. 577-82; discussion 582-4. | Outcome |
| Oshiro, B.T., et al., Decreasing elective deliveries before 39 weeks of gestation in an integrated health care system. Obstet Gynecol, 2009. 113(4): p. 804-11. | Population |
| Oshiro, B.T., et al., A multistate quality improvement program to decrease elective deliveries before 39 weeks of gestation. Obstet Gynecol, 2013. 121(5): p. 1025-31. | Population |
| Parikh, L., et al., Timing and consequences of early term and late term deliveries. J Matern Fetal Neonatal Med, 2014. 27(11): p. 1158-62. | Intervention |
| Torrance, E. and K. Dockery, Clinical governance and caesarean section. British Journal of Midwifery, 2003. 11(2): p. 94-96. | Control |
| Trojano, G., et al., The timing of elective caesarean delivery at term in lombardy: A comparison of 2010 and 2014. Italian Journal of Gynaecology and Obstetrics, 2016. 28(2): p. 48-51. | Intervention |
| Wilmink, F.A., et al., Timing of elective pre-labour caesarean section: A decision analysis. Aust N Z J Obstet Gynaecol, 2019. 59(2): p. 221-227. | Intervention |

Appendix C - Risk of bias assessment with ROBINS-I

Table 1 Risk of bias assessment with ROBINS-I

| **1.Bias due to confounding** | | | | | | | | |
| --- | --- | --- | --- | --- | --- | --- | --- | --- |
|  | **1.1 Is there potential for confounding of the effect of intervention in this study?**  If N/PN to 1.1: the study can be considered to be at low risk of bias due to confounding and no further signalling questions need be considered  If Y/PY to 1.1: determine whether there is a need to assess time-varying confounding: | **1.2. Was the analysis based on splitting participants’ follow up time according to intervention received?**  If N/PN, answer questions relating to baseline confounding (1.4 to 1.6)  If Y/PY, go to question 1.3. | Questions relating to baseline confounding only | | | Questions relating to baseline and time-varying confounding | | **Risk of bias judgement** |
|  |  |  | **1.4. Did the authors use an appropriate analysis method that controlled for all the important confounding domains?** | **1.5. If Y/PY to 1.4: Were confounding domains that were controlled for measured validly and reliably by the variables available in this study?** | **1.6. Did the authors control for any post-intervention variables that could have been affected by the intervention?** | **1.7. Did the authors use an appropriate analysis method that controlled for all the important confounding domains and for time-varying confounding?** | **1.8. If Y/PY to 1.7: Were confounding domains that were controlled for measured validly and reliably by the variables available in this study?** |  |
| Allen 2020 | Y | N | Y | Y | N | NA | NA | Moderate |
| Dunn 2013 | Y | N | N | NA | N | NA | NA | Critical |
| Gurol-Urganci 2014 | Y | N | PY | Y | N | NA | NA | Moderate |
| Hutcheon 2015 | Y | N | Y | Y | N | NA | NA | Moderate |
| MacAllister 2019 | Y | N | N | NA | N | NA | NA | Critical |
| Nicholl 2018 | Y | N | N | NA | N | NA | NA | Critical |
| Nicoll 2004 | Y | N | N | NA | N | NA | NA | Critical |
| Snowden 2016 | Y | N | Y | Y | N | NA | NA | Moderate |
| Tanger 2010 | Y | N | N | NA | N | NA | NA | Critical |
| Yamasato 2015 | Y | N | PN | NA | N | NA | NA | Serious |

| **2.Bias in selection of participants into the study** | | | | | | |
| --- | --- | --- | --- | --- | --- | --- |
|  | **2.1. Was selection of participants into the study (or into the analysis) based on participant characteristics observed after the start of intervention?**  If N/PN to 2.1: go to 2.4 | **2.2. If Y/PY to 2.1: Were the post-intervention variables that influenced selection likely to be associated with intervention?** | **2.3 If Y/PY to 2.2: Were the post-intervention variables that influenced selection likely to be influenced by the outcome or a cause of the outcome?** | **2.4. Do start of follow-up and start of intervention coincide for most participants?** | **2.5. If Y/PY to 2.2 and 2.3, or N/PN to 2.4: Were adjustment techniques used that are likely to correct for the presence of selection biases?** | **Risk of bias judgement** |
| Allen 2020 | N | NA | NA | Y | NA | Low |
| Dunn 2013 | N | NA | NA | Y | NA | Low |
| Gurol-Urganci 2014 | PY | N | NA | Y | NA | Moderate |
| Hutcheon 2015 | N | NA | NA | Y | NA | Low |
| MacAllister 2019 | N | NA | NA | Y | NA | Low |
| Nicholl 2018 | N | NA | NA | Y | NA | Low |
| Nicoll 2004 | N | NA | NA | Y | NA | Low |
| Snowden 2016 | N | NA | NA | Y | NA | Low |
| Tanger 2010 | N | NA | NA | Y | NA | Low |
| Yamasato 2015 | PN | NA | NA | Y | NA | Low |

| **3.Bias in classification of interventions** | | | | |
| --- | --- | --- | --- | --- |
|  | **3.1 Were intervention groups clearly defined?** | **3.2 Was the information used to define intervention groups recorded at the start of the intervention?** | **3.3 Could classification of intervention status have been affected by knowledge of the outcome or risk of the outcome?** | **Risk of bias judgement** |
| Allen 2020 | Y | Y | N | Low |
| Dunn 2013 | Y | Y | N | Low |
| Gurol-Urganci 2014 | Y | Y | N | Low |
| Hutcheon 2015 | Y | Y | N | Low |
| MacAllister 2019 | Y | Y | N | Low |
| Nicholl 2018 | Y | Y | N | Low |
| Nicoll 2004 | Y | Y | N | Low |
| Snowden 2016 | Y | Y | N | Low |
| Tanger 2010 | Y | Y | N | Low |
| Yamasato 2015 | Y | Y | N | Low |

| **4.Bias due to deviations from intended interventions** | | | | | | | |
| --- | --- | --- | --- | --- | --- | --- | --- |
|  | If your aim for this study is to assess the effect of assignment to intervention, answer questions 4.1 and 4.2 | | If your aim for this study is to assess the effect of starting and adhering to intervention, answer questions 4.3 to 4.6 | | | | **Risk of bias judgement** |
|  | **4.1. Were there deviations from the intended intervention beyond what would be expected in usual practice?** | **4.2. If Y/PY to 4.1: Were these deviations from intended intervention unbalanced between groups *and* likely to have affected the outcome?** | **4.3. Were important co-interventions balanced across intervention groups?** | **4.4. Was the intervention implemented successfully for most participants?** | **4.5. Did study participants adhere to the assigned intervention regimen?** | **4.6. If N/PN to 4.3, 4.4 or 4.5: Was an appropriate analysis used to estimate the effect of starting and adhering to the intervention?** |  |
| Allen 2020 | Y / PY / PN / N / NI | Y / PY / PN / N / NI | Y | NI | NI | NA | Low |
| Dunn 2013 | Y / PY / PN / N / NI | Y / PY / PN / N / NI | NI | NI | PN | N | Serious |
| Gurol-Urganci 2014 | Y / PY / PN / N / NI | Y / PY / PN / N / NI | Y | NI | NI | NA | Low |
| Hutcheon 2015 | Y / PY / PN / N / NI | Y / PY / PN / N / NI | Y | NI | NI | NA | Low |
| MacAllister 2019 | Y / PY / PN / N / NI | Y / PY / PN / N / NI | PN | NI | PN | N | Serious |
| Nicholl 2018 | Y / PY / PN / N / NI | Y / PY / PN / N / NI | NI | NI | NI | NA | No Information |
| Nicoll 2004 | Y / PY / PN / N / NI | Y / PY / PN / N / NI | NI | NI | NI | NA | No Information |
| Snowden 2016 | Y / PY / PN / N / NI | Y / PY / PN / N / NI | PN | NI | NI | NA | Moderate |
| Tanger 2010 | Y / PY / PN / N / NI | Y / PY / PN / N / NI | NI | NI | NI | NA | No Information |
| Yamasato 2015 | Y / PY / PN / N / NI | Y / PY / PN / N / NI | PN | NI | NI | N | Moderate |

| **5.Bias due to missing data** | | | | | | |
| --- | --- | --- | --- | --- | --- | --- |
|  | **5.1 Were outcome data available for all, or nearly all, participants?** | **5.2 Were participants excluded due to missing data on intervention status?** | **5.3 Were participants excluded due to missing data on other variables needed for the analysis?** | **5.4 If PN/N to 5.1, or Y/PY to 5.2 or 5.3: Are the proportion of participants and reasons for missing data similar across interventions?** | **5.5 If PN/N to 5.1, or Y/PY to 5.2 or 5.3: Is there evidence that results were robust to the presence of missing data?** | **Risk of bias judgement** |
| Allen 2020 | NI | NI | NI | NA | NA | No Information |
| Dunn 2013 | NI | NI | NI | NA | NA | No Information |
| Gurol-Urganci 2014 | N | PY | NI | NI | PY | Serious |
| Hutcheon 2015 | NI | NI | NI | NA | NA | No Information |
| MacAllister 2019 | NI | NI | NI | NA | NA | No Information |
| Nicholl 2018 | NI | NI | NI | NA | NA | No Information |
| Nicoll 2004 | NI | NI | NI | NA | NA | No Information |
| Snowden 2016 | PY | N | PN | NA | NA | Low |
| Tanger 2010 | NI | NI | NI | NA | NA | No Information |
| Yamasato 2015 | PN | PY | PN | NI | NI | Moderate |

| **6. Bias in measurement of outcomes** | | | | | | |
| --- | --- | --- | --- | --- | --- | --- |
|  | **Outcomes assessed** | **6.1 Could the outcome measure have been influenced by knowledge of the intervention received?** | **6.2 Were outcome assessors aware of the intervention received by study participants?** | **6.3 Were the methods of outcome assessment comparable across intervention groups?** | **6.4 Were any systematic errors in measurement of the outcome related to intervention received?** | **Risk of bias judgement** |
| Allen 2020 | CS rate <39+0 WG | N | PN | PY | N | Low |
| Dunn 2013 | CS rate <39+0 WG | N | Y | PY | N | Low |
| Gurol-Urganci 2014 | CS rate <39+0 WG | PN | N | PY | PN | Low |
| Hutcheon 2015 | CS rate <39+0 WG | N | Y | Y | N | Low |
| MacAllister 2019 | CS rate <39+0 WG | PN | Y | PY | PN | Low |
| Nicholl 2018 | CS rate <39+0 WG, NICU admission | Y | Y | Y | N | Moderate |
| Nicoll 2004 | CS rate <39+0 WG, NICU admission | Y | Y | Y | PN | Moderate |
| Snowden 2016 | CS rate <39+0 WG, NICU admission | Y | Y | Y | PY | Serious |
| Tanger 2010 | CS rate <39+0 WG | N | Y | PY | PN | Low |
| Yamasato 2015 | CS rate <39+0 WG | N | Y | PY | PN | Low |

| **7. Bias in selection of the reported result** | | | | |
| --- | --- | --- | --- | --- |
|  | **Is the reported effect estimate likely to be selected, on the basis of the results, from...** | | |  |
|  | **7.1. ... multiple outcome *measurements* within the outcome domain?** | **7.2 ... multiple analyses of the intervention-outcome relationship??** | **57.3 ... different subgroups?** | **Risk of bias judgement** |
| Allen 2020 | PN | PN | PN | Moderate |
| Dunn 2013 | N | N | N | Low |
| Gurol-Urganci 2014 | N | N | N | Low |
| Hutcheon 2015 | N | PY | N | Moderate |
| MacAllister 2019 | N | N | N | Low |
| Nicholl 2018 | N | N | N | Low |
| Nicoll 2004 | N | N | N | Low |
| Snowden 2016 | N | N | N | Low |
| Tanger 2010 | N | N | N | Low |
| Yamasato 2015 | N | N | N | Low |
